# High-Temperature Fiber-Optic Fabry–Perot Vibration Sensor Based on Single-Crystal Sapphire

**DOI:** 10.3390/s23104952

**Published:** 2023-05-21

**Authors:** Hua Liu, Pinggang Jia, Chengxin Su, Aihao Zhao, Jia Liu, Qianyu Ren, Jijun Xiong

**Affiliations:** State Key Laboratory of Dynamic Measurement Technology, North University of China, Taiyuan 030051, China

**Keywords:** fiber-optic sensor, Fabry–Perot, high-temperature applications, vibration measurement, single-crystal sapphire

## Abstract

In this paper, a fiber-optic Fabry–Perot (F–P) vibration sensor that can work at 800 °C is proposed. The F–P interferometer is composed of an upper surface of inertial mass placed parallel to the end face of the optical fiber. The sensor was prepared by ultraviolet-laser ablation and three-layer direct-bonding technology. Theoretically, the sensor has a sensitivity of 0.883 nm/g and a resonant frequency of 20.911 kHz. The experimental results show that the sensitivity of the sensor is 0.876 nm/g in the range of 2 g to 20 g at an operating frequency of 200 Hz at 20 °C. The nonlinearity was evaluated from 20 °C to 800 °C with a nonlinear error of 0.87%. In addition, the z-axis sensitivity of the sensor was 25 times higher than that of the *x*-axis and *y*-axis. The vibration sensor will have wide high-temperature engineering-application prospects.

## 1. Introduction

Vibration sensors have a wide range of applications in earthquake monitoring, tsunami monitoring, aero engines, geological exploration and nuclear reactors [1,2,3,4]. At present, there are several types of vibration sensor, such as piezoelectric [5,6], piezoresistive [7], capacitive [8] and fiber-optic vibration sensors [9]. However, vibration sensors are often exposed to harsh environments, such as high temperatures and corrosion, in the application areas listed above. The performance of electrical vibration sensors is greatly affected by temperature [10,11]. Compared with electrical vibration sensors, fiber-optic vibration sensors have been widely studied for their immunity to electromagnetic interference, flexibility and high temperature resistance [12,13].

In recent years, there has been increasing interest in the development of high-temperature fiber-optic vibration sensors. At present, fiber-optic vibration sensors are mainly manufactured by mechanical processing [14], MEMS [15] and laser-processing technology [16]. Jia et al. [17] proposed a temperature-compensated fiber-optic F–P accelerometer with a mechanically processed cantilever-beam structure, with a nonlinearity of approximately 1.03%. The standard deviation of the phase sensitivity is approximately 0.002 rad/g in the temperature range of 30 °C to 90 °C. Qian et al. [18] demonstrated an extrinsic high-temperature fiber-optic F–P vibration sensor based on MEMS technology, which has a sensitivity of 2.48 nm/g at 20 °C. The nonlinearity of the sensor at 300 °C is 1.88%. For higher-temperature applications, high-temperature-resistant materials, such as silicon carbide and sapphire, are receiving increasing attention [19]. Cui et al. [20] proposed a sapphire fiber-optic high-temperature vibration sensor. The sensitive unit was prepared with a femtosecond laser. The sensor-output error at 25 °C, 300 °C and 600 °C does not exceed 1% of full scale. Huang et al. [21] prepared a F–P vibration sensor using a silicon-carbide substrate and fiber-optic sapphire. The nonlinearity of the sensor is 4.96% at 800 °C. However, these sensors are difficult to prepare in batches with high consistency.

In this paper, a high-temperature fiber-optic vibration sensor based on single-crystal sapphire is proposed. The sensor uses a gold-coated single-mode fiber (GSMF, Fiberguide, NJ, USA) for vibration-signal reception and transmission. The GSMF can not only protect quartz optical fibers, but also withstand high temperatures of 800 °C. The F–P cavity is composed of an upper surface of inertial mass placed parallel to the end face of the optical fiber. The sensitive unit was prepared by using the ultraviolet-laser method. The sensor was directly bonded by three-layer-sapphire direct-bonding technology, which can achieve high consistency in batch preparation. The sapphire-vibration-sensitive units with different parameters were analyzed and compared by using a numerical analysis. Through these methods, the sensor testing system was established, the sensor was tested and the experimental results were analyzed.

## 2. Sensor’s Working Principle

**Figure 1:**
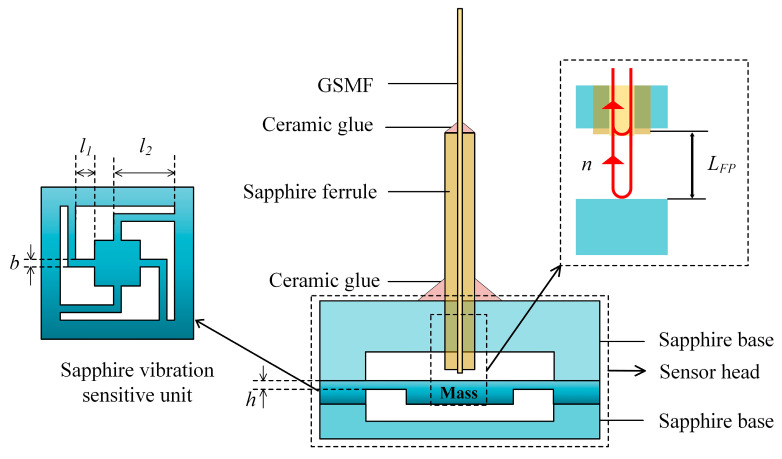
Schematic diagram of the sapphire-vibration sensor.

The structure of the sensor is shown in Figure 1. The fiber-optic F–P high-temperature vibration sensor consisted of a GSMF, a sapphire ferrule, a sapphire-vibration-sensitive unit and sapphire bases. The sensor head was prepared by three-layer-sapphire direct-bonding technology. The sensitive unit was composed of an inertial mass and a multi-cantilever-beam structure. The vibration-sensitive unit is a center-symmetric structure that is stable, not suitable for torsion and easy to machine. The lower surface of the vibration-sensitive unit was roughened. The multi-cantilever-beam structure was prepared by ultraviolet-laser-ablation technology. The F–P cavity consisted of the end of the optical fiber and the upper surface of the inertial mass, and the light-transmission direction was perpendicular to the upper surface of the inertial mass. The light emitted from the amplified spontaneous emission (ASE) source was injected into the F–P cavity through the fiber-optic, creating multi-beam interference. When the acceleration changed, the inertial mass moved relative to the end face of the optical fiber due to inertial forces, so the F–P’s cavity length L_FP_ changed with the acceleration signal. Next, the acceleration change was converted into a phase change in the interference signal. The relationship between the phase change of the interference signal and the F–P’s cavity-length change can be expressed as [22]:

(1) $$\Delta \varphi (t)=\frac{4\pi n}{\lambda }\Delta L_{FP}(t),$$

where *n* represents the refractive index of air, and λ represents the light wavelength. The *L_FP_* change caused by the axial acceleration change is numerically equal to the axial displacement of the mass.

Due to the low reflectivity of the end face of optical fiber and the upper surface of the inertial mass, multi-beam interference can be approximated as double-beam interference. The double-beam-interference equation is expressed as:

(2) $$\frac{I^{\left(r\right)}}{I^{\left(i\right)}}=R_{1}+R_{2}-2\sqrt{R_{1}R_{2}}\cos\varphi ,$$

where 𝐼^(𝑟)^ represents the intensity of the reflected light, 𝐼^(𝑖)^ represents the intensity of the incident light, 𝑅_1_ and 𝑅_2_ represent the reflectance of the two reflective surfaces, respectively, and *φ* represents the phase of the light signal.

Under acceleration, the center line of the cantilever beam is curved into a continuous smooth planar curve. Neglecting the lateral load of the cantilever beam and deformation of the inertial mass, the maximum deflection ΔY of the cantilever beam (the axial displacement of the inertial mass) is expressed as [17]:

(3) $$\Delta Y=-\frac{mal^{3}}{4Ebh^{3}},$$

where m represents the mass of the inertial mass, a represents the axial acceleration, E represents the Young’s modulus of sapphire, l, b and h represent the length, width and height of the cantilever beam, respectively, while ${l=l}_{1}+l_{2}$. According to Equation (3), the sensitivity of the sensor is expressed as:

(4) $$S=\frac{mgl^{3}}{4Ebh^{3}}.$$

The resonance frequency of the sensitive unit can be given by [21]:

(5) $$f=\frac{1}{\pi }\sqrt{\frac{Ebh^{3}}{m_{0}l^{3}}}.$$

From Equations (4) and (5), it can be seen that the sensitivity and resonant frequency of the sensitive unit show opposite trends when the dimensions of the vibration-sensitive-unit change. The shorter the cantilever-beam length (l), the thicker the cantilever-beam thickness (h) and the wider the cantilever-beam width (b), the higher the resonant frequency of the sensitive unit and the lower the sensitivity. The sensor size, sensitivity, resonance frequency and other characteristics need to be considered in the design process. The sensitive-unit-structure parameters are shown in Table 1. From Equations (4) and (5), the sensitivity of the sensor in this paper is 0.883 nm/g and the resonant frequency is 20.911 kHz.

The vibration-sensitive unit was manufactured with a single crystal sapphire, which had a coefficient of thermal expansion of 5.8 × 10^−6^/°C, a refractive index of 1.768 and a very high melting point (2030 °C). At the same time, the single-crystal sapphire had good thermal conductivity, mechanical strength, electrical insulation, thermal stability and optical properties and, furthermore, it is often used as an excellent high-temperature-resistant material for a wide range of optical applications [23,24].

The sensitivity and resonant frequency of the sensor change with external temperature due to changes in material properties. The influence of temperature on the sensor was analyzed through the simulation. The temperature compensation of the sensor can be directed through the simulation results.

**Figure 2:**
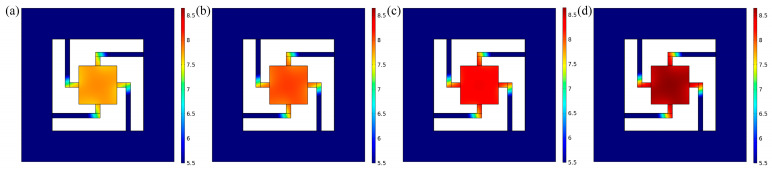
Displacement distribution of vibration-sensitive unit at different temperatures under 10 g of acceleration. (**a**) 200 °C. (**b**) 400 °C. (**c**) 600 °C. (**d**) 800 °C.

The finite-element-simulation analysis of the sensitive element was performed at 200 °C, 400 °C, 600 °C and 800 °C. The displacement distribution of the sensitive unit under 10 g of acceleration at different temperatures is shown in Figure 2. The displacement curve of the maximum displacement of the vibration-sensitive-unit center at different temperatures is shown in Table 2. It can be seen that the displacement of the sensor under 10 g of acceleration increased as the temperature increased.

## 3. Sensitive-Unit Preparation

Due to the high temperature resistance, difficult corrosion and high level of hardness of sapphire materials, there are only a few ways to etch sapphire, such as mechanical cutting [25], wet etching [26], dry etching [27] and laser ablation [28]. Compared with other processing methods, laser ablation is more suitable for processing hard and brittle materials, with the advantages of fast processing speed and flexible processing methods [29].

**Figure 3:**
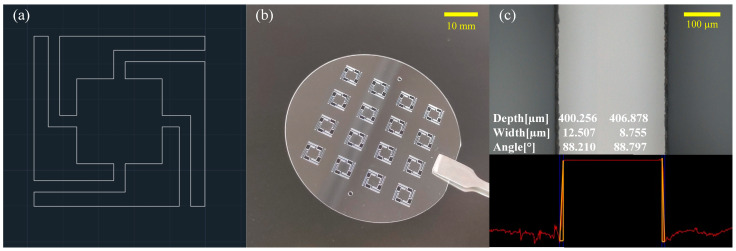
Ultraviolet-laser array for ablation of sapphire. (**a**) Laser path. (**b**) Photograph of sensitive unit array. (**c**) Confocal microscope photograph of the ultraviolet-laser-etched boundary.

The laser-ablation experiments [29,30] were set up using the ultraviolet micromachining system (DelphiLaser FPC03, Suzhou, China) to select the appropriate laser-processing parameters. In the experiments, an ultraviolet laser with a wavelength of 355 nm was used for the ablation of the sapphire. The ablation experiments were performed on the sapphire by using different parameters: pulse width, laser power, working pulse frequency, scanning speed and spotting time. The edge quality, depth and taper of the microgroove obtained from the experiment were examined and analyzed. Combining the experimental results, the parameters shown in Table 3 were selected for the preparation of the sensitive unit. The laser path used to prepare the sensitive unit is shown in Figure 3a. The prepared array of sensitive units is shown in Figure 3b, which proves that the sensor is suitable for mass production.

The edges of the sensitive unit completed by the ultraviolet-laser ablation were magnified and measured using a confocal microscope (Olympus LEXTOLS4100, Japan). As shown in Figure 3c, the verticality of both sides of the ablation sidewall was measured. The angles of the left sidewall and right sidewall were 88.210 deg and 88.797 deg, respectively. As shown in Figure 3, the etched edges had good consistency and uniform width, which helped to reduce the machining errors of the multi-cantilever-beam and inertial mass dimensions. In addition, some of the ablation and protrusion at the etched edges can be removed with simple cleaning, which ensures the cleanliness and flatness of the sapphire wafers.

**Figure 4:**
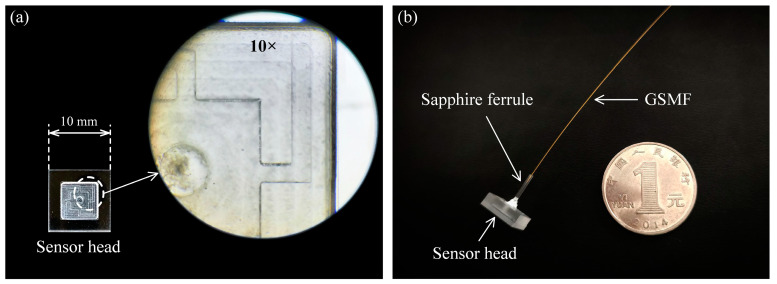
(**a**) Photograph of the sensor head. (**b**) Photograph of the sapphire-vibration sensor.

The sapphire bases were processed by using computer numerical control (CNC) technology. The sensitive unit and sapphire bases were directly bonded by using three-layer-sapphire direct-bonding technology (the bonding temperature was about 1100 °C and the bonding pressure was about 5 MPa), as shown in Figure 4a. The three-layer-sapphire direct-bonding technology was used to achievee batch preparation with high consistency in wafer manufacturing and laid the foundation for the engineering. The GSMF, sapphire ferrule and sensor head were integrated with ceramic glue. The prepared sapphire-vibration sensor is shown in Figure 4b.

## 4. Experiments and Analysis

**Figure 5:**
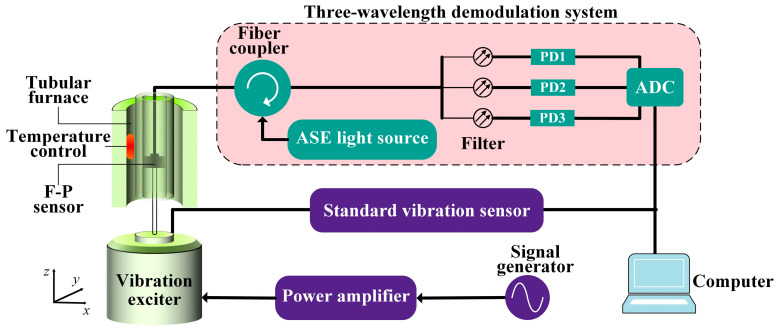
Sapphire-vibration-sensor high-temperature testing system.

**Figure 6:**
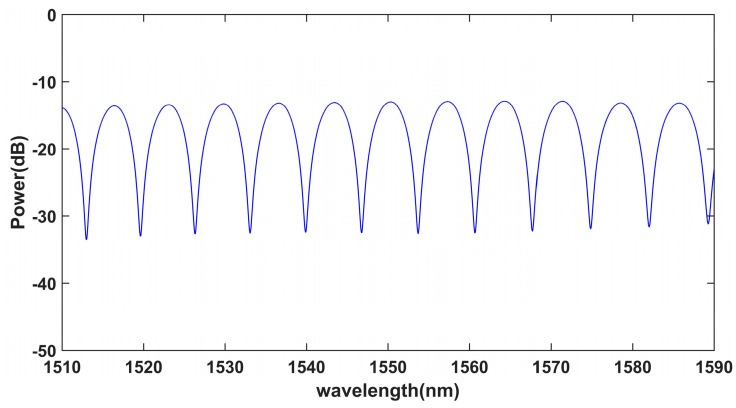
Spectrum of the sapphire-vibration sensor.

A high-temperature vibration-testing system was built to test the performance of the sensor, as shown in Figure 5. The system consisted of a vibration-excitation system, a tubular heating furnace, a standard vibration sensor and a three-wavelength demodulation system [31]. The sensor was connected to the vibration exciter using a stub and placed in a tubular heating furnace. A computer was used to control the signal generator and power amplifier to provide the alternating current signal for the vibration exciter. The sensor output was connected to a demodulation system and the output signal was displayed on the computer. A standard accelerometer was set on the vibration exciter to monitor the acceleration signal in real time and to transmit and display it on the computer. The spectrum of the sapphire-vibration sensor is shown in Figure 6. According to Figure 1 and Figure 6, the F–P cavity length of the sensor was 173.287 µm. That is, the distance between the end face of the optical fiber and the upper surface of the inertial mass was 173.287 µm.

**Figure 7:**
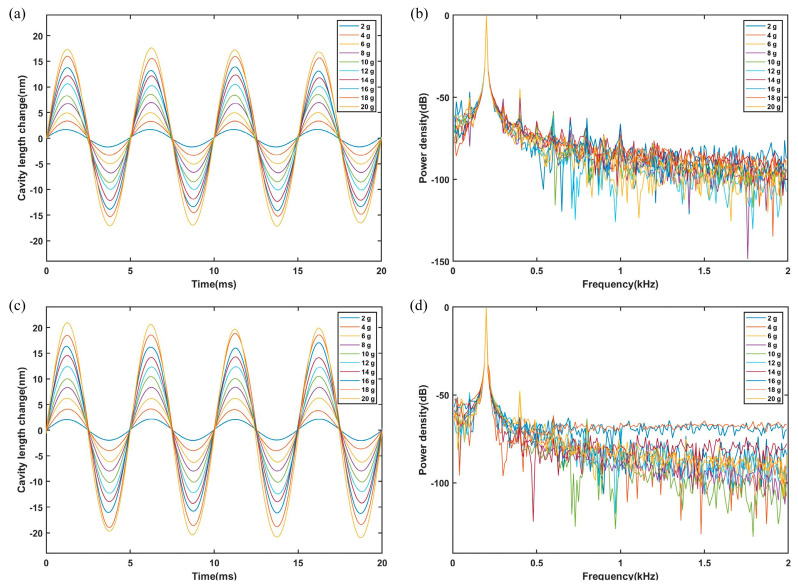
The sensor output from 2 g to 20 g at 200 Hz. (**a**) Sensor waveform at 20 °C. (**b**) Power-density spectrum at 20 °C. (**c**) Sensor waveform at 800 °C. (**d**) Power-density spectrum at 800 °C.

The sapphire-vibration sensor was tested at room temperature (20 °C), 200 °C, 400 °C, 600 °C and 800 °C, respectively. The tests were conducted at each temperature with accelerations ranging from 2 g to 20 g at 2-g intervals and the frequency was 200 Hz. Each vibration experiment was conducted for 1 min before collecting data when the acceleration was stable. Figure 7a,c show the vibration waveforms of the sensor after low-pass filtering at 20 °C and 800 °C, respectively. According to the experimental results, the sensitivities of the sensor were 0.876 nm/g (20 °C) and 1.024 nm/g (800 °C), respectively. The standard evaluation of the sensitivity was 0.008 nm/g. Figure 7b,d show a diagram of the power-density spectrum of the sensor.

**Figure 8:**
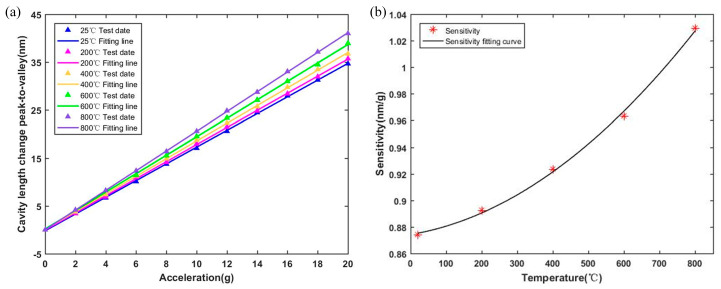
(**a**) Cavity-length-change peak to valley of the sensor at different temperatures. (**b**) The temperature cross-sensitivity of the sensor.

The experimental results of the sapphire-vibration sensor were evaluated over the temperature range of 20 °C to 800 °C. The cavity-length change peak-to-valley values were calculated and a least-squares linear fit was made. As shown in Figure 8a, the cavity-length-change peak-to-valley values of the sensor were approximately linear with acceleration at each temperature, which indicated that the sapphire-vibration sensor maintained the characteristics of a second-order system below 800 °C. The cavity-length-change peak-to-valley values were evaluated from 20 °C to 800 °C with a nonlinear error of 0.87%. The standard uncertainty of the nonlinear error was 0.25%. The sensitivities of the sensor at different temperatures are shown in Figure 8b. As the temperature increased, the sensitivity of the sensor gradually increased, due to the influence of the temperature on parameters such as the Young’s modulus and the thermal expansion coefficient of sapphire.

**Figure 9:**
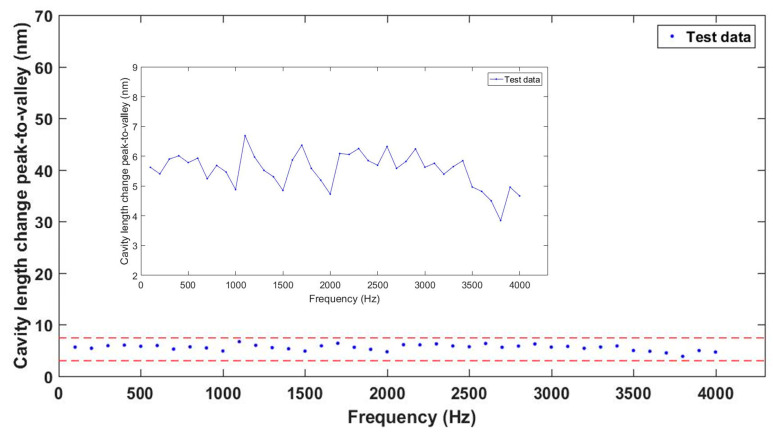
Cavity-length-change peak-to-peak values of the sensor versus excitation frequency.

In order to test the frequency response of the sensor, frequency-sweep experiments were performed. The acceleration was set to 6 g at room temperature (20 °C) and the operating frequencies were set in the range of 100 Hz to 4000 Hz at 100-Hz intervals. The cavity-length-change peak-to-valley values of the sensor are shown in Figure 9. The experimental results show that under different operating frequencies and with the same acceleration applied to the sensor, the maximum fluctuation was 1.89 g. which indicates that the frequency range was much smaller than the resonant frequency of the sensor; the sensor can work stably in a frequency range of 100–4000 Hz.

**Figure 10:**
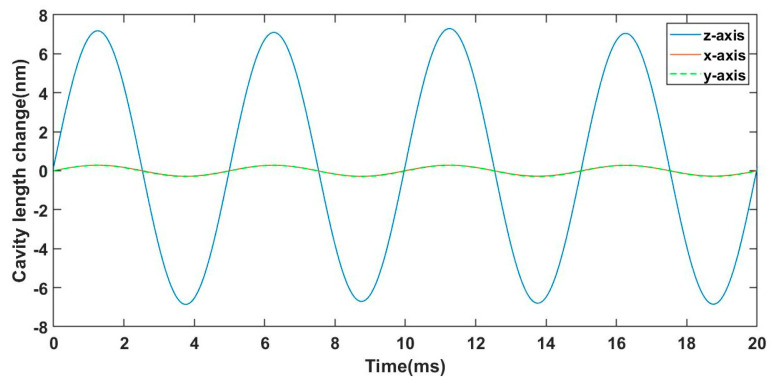
The vibration waveforms of the sensor in three directions.

Experiments on the directional cross-sensitivity of the sensor were performed. The sensor was fixed on the vibration exciter along the *x*-axis, *y*-axis and *z*-axis, respectively. The acceleration was set to 10 g and the frequency was 200 Hz. The vibration waveforms of the sensor in three directions are shown in Figure 10. The experimental results show that the cavity-length-change peak-to-valley value of the sensor on the *z*-axis was 25 times higher than those on the *x*-axis and *y*-axis. In addition, the vibration waveforms in the other two directions approximately overlapped. Therefore, it can be concluded that the cantilever beam hardly twists when the sensitive unit is subjected to acceleration in the axial direction.

## 5. Conclusions

In this paper, a fiber-optic Fabry–Perot vibration sensor that can work at a high temperature of 800 °C high was proposed. The preparation and testing of the sapphire vibration sensor were completed. The sensitive unit was produced by using ultraviolet-laser-ablation technology and the sensor head consisted of the sensitive unit and the sapphire bases using the three-layer-sapphire direct-bonding technology. Therefore, the sapphire-vibration sensor proposed in this paper has the ability to produce wafer-level batches and high consistency. Finally, the sensor consists of a GSMF, a sapphire ferrule and a sensor head. The results of the experiment on the high-temperature vibration indicate that the sensor can operate in the temperature range of room temperature (20 °C) to 800 °C. The nonlinearity of the sensor at 800 °C is 0.87%, which proves the sensor’s good linear response to acceleration at high temperatures. The results of the frequency-sweep experiments show that the sensor can work up to 4 kHz. In addition, the *z*-axis sensitivity of the sensor is 25 times higher than its sensitivities on the other two axes (*x*-axis and *y*-axis). These sapphire-vibration sensors, which can be prepared in batches with high consistency, will have a wide range of engineering applications in the field of vibration measurement in high-temperature environments.

## Figures and Tables

**Table 1:** Vibration-sensitive-unit-structure parameters.

Parameters	Symbol	Value
Length of cantilever beam/mm	l	3.8
Width of cantilever beam/mm	b	0.3
Thickness of cantilever beam/mm	h	0.2
Density of sapphire/kg·m^−3^	ρ	3980
Mass of inertial mass/kg	$m_{0}$	$4.21\times 10^{-6}$
Young’s modulus of sapphire/GPa	E	497
Sensitivity/nm·g^−1^	S	0.883
Frequency/Hz	*f*	20,911

**Table 2:** The maximum displacement of the center of the vibration-sensitive unit at different temperatures under 10 g of acceleration.

Temperature/°C	200	400	600	800
Displacement /nm	7.87	8.10	8.35	8.63

**Table 3:** Ultraviolet-laser-preparation parameters.

Symbol	Pulse Width/ns	Laser Power/W	Working Pulse Frequency/kHz	Scanning Speed/mm·s^−1^	Spotting Time/ms
Value	11	6.60	30	0.5	1000

## Data Availability

Data will be made available on request.
